# Timing for maximum anaesthetic effect of topical cream during early infant circumcision (EIC) in Rakai, Uganda

**DOI:** 10.1002/bco2.223

**Published:** 2023-01-16

**Authors:** Stephen Kiboneka, Aggrey Anok, Regina Nakabuye, Silas Odiya, Julius Magembe, Rose Nazziwa, Charles Ddamulira, Andrew Mulooki, Ronald Moses Galiwango, Stephen Watya, Philip S. Li, Richard K. Lee, Ronald H. Gray, Godfrey Kigozi, Edward Nelson Kankaka

**Affiliations:** ^1^ Rakai Health Sciences Program Kalisizo Uganda; ^2^ Weill Cornell Medicine of Cornell University New York New York USA; ^3^ Urocare Hospital Kampala Uganda; ^4^ Johns Hopkins Bloomberg School of Public Health Baltimore Maryland USA

**Keywords:** EIC, EIMC, infant circumcision, pain measurement, timing, topical anaesthesia

## Abstract

**Objectives:**

The objective of this study is to determine the optimal timing for device‐based infant circumcision under topical anaesthesia.

**Subjects/patients:**

We include infants aged 1–60 days who were enrolled in a field study of the no‐flip ShangRing device at four hospitals in the Rakai region of south‐central Uganda, between 5 February 2020 and 27 October 2020.

**Methods:**

Two hundred infants, aged 0–60 days, were enrolled, and EMLA cream was applied on the foreskin and entire penile shaft. The anaesthetic effect was assessed every 5 min by gentle application of artery forceps at the tip of the foreskin, starting at 10 min post‐application until 60 min, the recommended time to start circumcision. The response was measured using the Neonatal Infant Pain Scale (NIPS). We determined the onset and duration of anaesthesia (defined as <20% of infants with NIPS score >4) and maximum anaesthesia (defined as <20% of infants with NIPS score >2).

**Results:**

Overall, NIPS scores decreased to a minimum and reversed before the recommended 60 min. Baseline response varied with age, with minimal response among infants aged 40 days. Overall, anaesthesia was achieved after at least 25 min and lasted 20–30 min. Maximum anaesthesia was achieved after at least 30 min (except among those aged >45 days where it was not achieved) and lasted up to 10 min.

**Conclusion:**

The optimal timing for maximum topical anaesthesia occurred before the recommended 60 min of waiting time. A shorter waiting time and speed may be efficient for mass device‐based circumcision.

## 1 INTRODUCTION

Male circumcision (MC) was recommended by the World Health Organization (WHO) and the Joint United Nations Program on HIV/AIDS (UNAIDS) as part of combination HIV prevention interventions in countries with low MC prevalence and high HIV burden^1^ after three circumcision trials demonstrated 60% effectiveness in reducing female to HIV transmission.^2^, ^3^, ^4^ National HIV programs and donor agencies have primarily targeted MC scale‐up adolescents and adults for quicker impact^5^ and focussed much less on infants. However, infant circumcision is technically more accessible and cheaper, healing is faster, there is no risk of early sex resumption, and it may be more sustainable in the long term.^6^, ^7^, ^8^

Early infant circumcision (EIC) can be performed by non‐physician health workers using devices and topical anaesthetics,^9^, ^10^ removing the need for an injection. When using topical anaesthetics, the WHO recommends circumcision 60 min after application of the anaesthetic.^11^ However, there are reports of incomplete pain control during infant circumcision,^12^ and there is minimal literature on the optimal timing of the circumcision after the administration of anaesthesia. Also, although infant pain can be measured using standard tools such as the Neonatal Infant Pain Scale (NIPS),^13^ pain responses can be variable and little is known about what determines the level of pain sensitivity or pain‐like reactions in infants.

We assessed the optimal timing of infant circumcision after administration of topical anaesthesia. The success of infant circumcision programs is largely dependent on maintaining both the ease of the procedure and adequate pain control.

## 2 SUBJECTS AND METHODS

### 2.1 Study design and setting

We conducted a short observational substudy during a non‐comparative field study of no‐flip ShangRing infant circumcisions performed by non‐physician providers in routine clinical settings. This field study followed a clinical trial that comparing the safety and acceptability of infant circumcisions performed by non‐physician health workers using the no‐flip ShangRing device versus the Mogen clamp (clinicaltrials.gov identifier NCT03338699). The field study was simultaneously conducted at three sites at Iringa Regional Referral Hospital in Tanzania, Homa Bay County Teaching and Referral Hospital in Kenya, and the Rakai Health Sciences Program in Uganda. The observational substudy presented here was only conducted in Uganda at four facilities in the Rakai region (Kalisizo hospital, Masaka hospital, Lyantonde Hospital, and Rakai hospital), coordinated at the Rakai Health Sciences Program in Kalisizo, south‐central Uganda (latitude −0.53, longitude 31.62).

### 2.2 Selection of participants

We included all eligible infants whose parents or legally acceptable representatives (LAR) provided informed consent for their infants to participate in the field study at the four facilities in Rakai. Eligibility included healthy infant boys from 24 h up to 60 days of age, born after ≥ 37 weeks of gestation, weighing ≥ 2.5 kg, with no penile abnormality, no family history of bleeding disorders, and a history of maternal or infant Tetanus vaccination. Infants were enrolled between 5 February 2020 and 27 October 2020.

### 2.3 Primary outcome

The primary outcome of interest was the NIPS score measured at 5‐min intervals before circumcision, that is, between 10 min after application of a topical analgesic cream containing lidocaine and prilocaine (Eutectic Mixture of Local anaesthetics) and before performing the circumcision with the ShangRing at 60 min. Sensitivity to pain was tested by gentle application of toothed artery forceps at the foreskin. The pain was measured using the NIPS scale,^13^ a standard tool commonly used for pain measurement in infants. The NIPS scale is a behavioural scale composed of six indicators of infant pain or distress. These include facial expression, cry, breathing patterns, arms, legs, and state of arousal. Each is scored 0 or 1, except cry, which may be scored 0, 1, or 2. A total score between 0 and 2 is considered mild pain to no pain, a score of 3–4 as mild to moderate pain, and 4–7 as severe pain (Table 1). We also retrospectively obtained and appended the NIPS scores during circumcision to the NIPS scores before circumcision. For example, intraoperative measurements at time points between 60 and 65 min after application of topical anaesthesia were assigned to the 65th minute, and so on (each infant had one intraoperative measurement). The circumcisions lasted a median of 11 min (IQR 10–13).

**TABLE 1:** The Neonatal Infant Pain Scale

Pain Assessment Tools		
Neonatal/Infant Pain Scale (NIPS)		
Recommended for Children less than 1 year old – A score greater than 3 indicates pain		
Pain assessment		Score
Facial expression		
0‐Relaxed muscles	Restful face, neutral expression	
1‐Grimace	Tight facial muscles; furrowed brow, chin, jaw, (negative facial expression – nose, mouth and brow)	
Cry		
0‐No Cry	Quiet, not crying	
1‐Whimper	Mild moaning, intermittent	
2‐Vigorous cry	Loud scream; rising, shrill, continuous (Note: Silent cry may be scored if baby is intubated as evidenced by obvious mouth and facial movement.)	
Breathing patterns		
0‐Relaxed	Usual pattern for this infant	
1‐Change in breathing	Indrawing, irregular, faster than usual; gagging; breath holding	
Arms		
0‐Relaxed/Restrained	No muscular rigidity; occasional random movement of arms	
1‐Flexed/Extended	Tense, straight arms; rigid and/or rapid extension, flexion	
Legs		
0‐Relaxed/Restrained	No muscular rigidity; occasional random leg movement	
1‐Flexed/Extended	Tense, straight legs; rigid and/or rapid extension, flexion	
State of arousal		
0‐Sleeping/Awake	Quiet, peaceful sleeping or alert random leg movement	
1‐Fussy	Alert, restless, and thrashing	

*Notes*: The NIPS^13^ was developed at Children's Hospital of Eastern Ontario. The NIPS assesses six behavioural indicators in response to painful procedures in preterm newborns (gestational age < 37 weeks) and full‐term newborns (gestational age > 37 weeks to 6 weeks after delivery). Table adapted from the Pain Assessment and Management Initiative (https://pami.emergency.med.jax.ufl.edu/wordpress/files/2019/10/Neonatal‐Infant‐Pain‐Scale‐NIPS.pdf).

### 2.4 Explanatory variables

The main explanatory variable was time after application of topical anaesthetic cream. We also captured infant age to assess differential pain responses/pain experience by age, if any and the recruiting health facility.

### 2.5 Analysis

**FIGURE 1:**
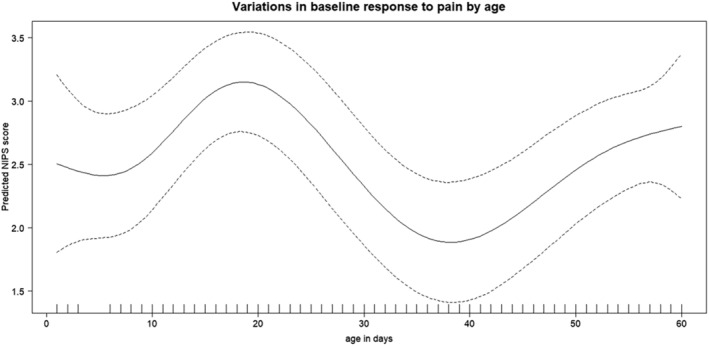
Variations in baseline responses to gentle application of forceps, by age. The dotted lines represent 95% confidence intervals.

Infant characteristics were described using proportional distributions for categorical variables. We assessed for baseline variations in pain responses with respect to age using a generalized additive model to determine the need for stratification by age in case of a non‐linear relationship. Baseline measurements were the first two NIPS scores at 10 and 20 min after the topical cream application. After observing non‐linear variations (Figure 1), we grouped infants into those aged 0–15 days, 16–30 days, 31–45 days, and 46–60 days. In each age group, we calculated and plotted the prevalence and 95% confidence interval of pain (NIPS score > 2) and the prevalence and confidence interval of severe pain (NIPS score > 4) at each 5‐min interval before and during circumcision (all infants included had a measurement at each of these time points).

We then determined the time points at which anaesthesia was achieved (defined as less than 20% of infants with NIPS scores > 4; the confidence interval also entirely falling below 20% prevalence). We also determined the time points at which maximum anaesthesia was achieved (defined as less than 20% of infants with NIPS scores > 2; the confidence interval also entirely falling below 20% prevalence, which we considered as ‘virtual elimination’ of pain). In secondary analyses, we assessed the temporal variation in median NIPS scores, which are not sensitive to outliers if present.

### 2.6 Ethical considerations

The field study under which these observations were performed was approved by the Uganda Virus Research Ethics Committee (UVRI‐REC) and the Uganda National Council for Science and technology in Uganda as well as the Weill Medical College of Cornell University Institutional Review Board. Application of forceps for evaluation of anaesthetic effect was gentle and performed before circumcision in the presence of the mother. Infants with detectable responses were soothed by breastfeeding prior to circumcision. All infants received rectal paracetamol prior to circumcision, sucrose solution during circumcision in case of any response to enable completion of the procedure,^12^, ^14^ and oral paracetamol after circumcision.

## 3 RESULTS

### 3.1 Infant characteristics

Measurements were done for a total of 200 infants (sufficient to detect pain in at least 20% of infants with 88% power and 95% confidence): 56 infants (28.0%) were enrolled at Kalisizo hospital, 40 (20.0%) at Lyantonde hospital, 59 (29.5%) at Masaka hospital, and 45 (22.5%) at Rakai hospital. Age was roughly uniformly distributed between 0 and 60 days (Table 2).

**TABLE 2:** Infant characteristics

Characteristic	*n* (%)
Age in days	
0–15	46 (0.23)
16–30	48 (0.24)
31–45	38 (0.19)
46–60	68 (0.34)
Recruiting facility	
Kalisizo Hospital	56 (0.28)
Lyantonde Hospital	40 (0.20)
Masaka Hospital	59 (0.30)
Rakai Hospital	45 (0.23)

### 3.2 Variations in baseline response by age

We observed a nonlinear relationship with a bimodal distribution of NIPS scores taken between 10 and 20 min after EMLA application, a peak at about 30 days and a minimum at about 40 days of age (Figure 1).

### 3.3 Onset and duration of anaesthesia

**FIGURE 2:**
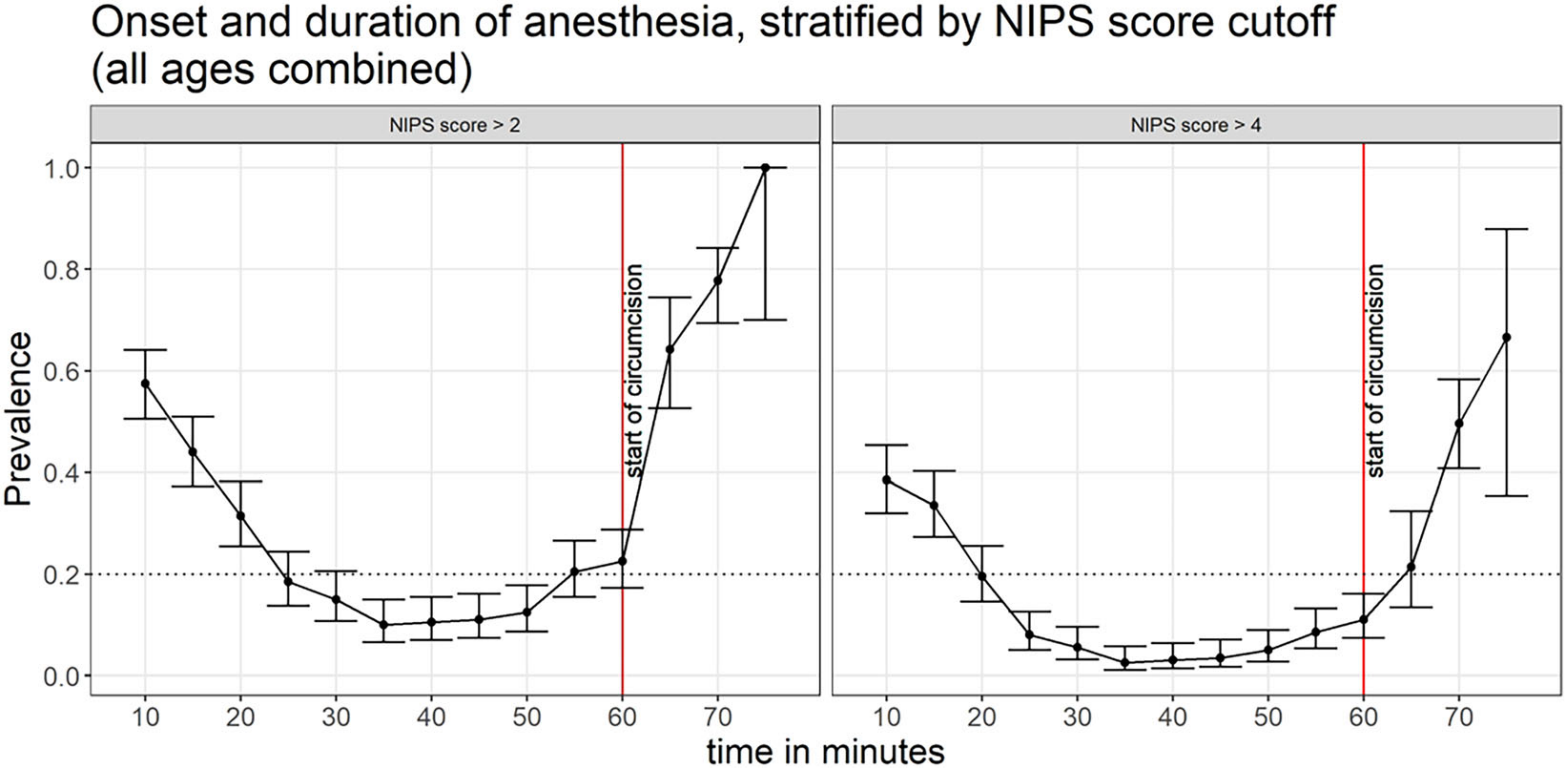
Proportions with NIPS score > 2 or NIPS score > 4 following application of topical anaesthetic cream, all ages combined. The error bars represent 95% confidence intervals.

**FIGURE 3:**
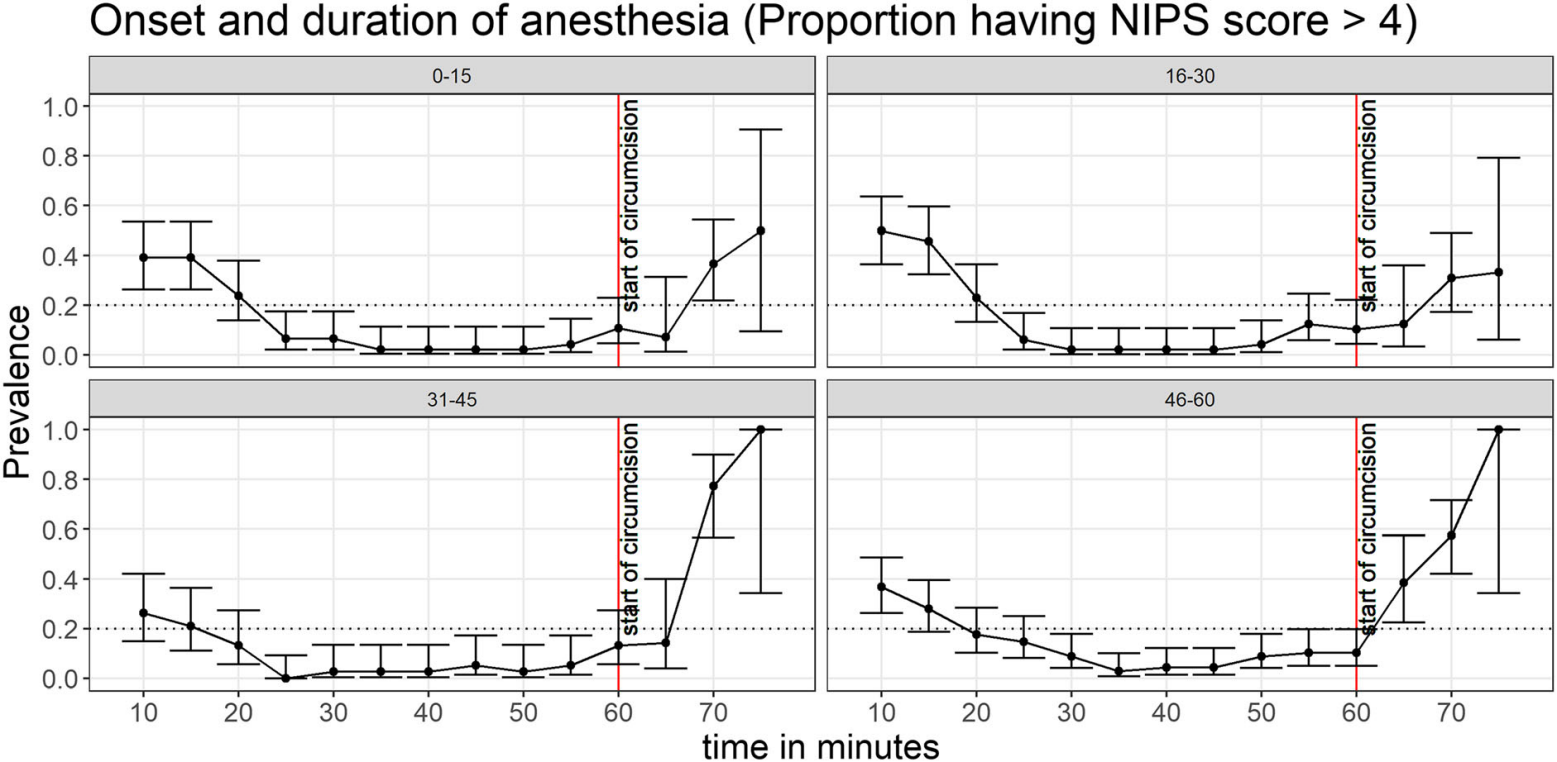
Proportions with NIPS score > 4 following application of topical anaesthetic cream, stratified by age. The error bars represent 95% confidence intervals.

Overall (all ages combined), NIPS scores > 4 were detectable in a few infants (<20%) after at least 20 min following topical anaesthetic application, with anaesthesia lasting <45 min (Figure 2). Stratifying by age, the onset of anaesthesia was delayed at 30 min among those aged 46–60 days. The duration of anaesthesia varied with age, lasting 30, 25, 30, and 20 min among those aged 1–15, 16–30, 31–45, and 46–60 days, respectively (Figure 3).

### 3.4 Onset and duration of maximum anaesthesia

**FIGURE 4:**
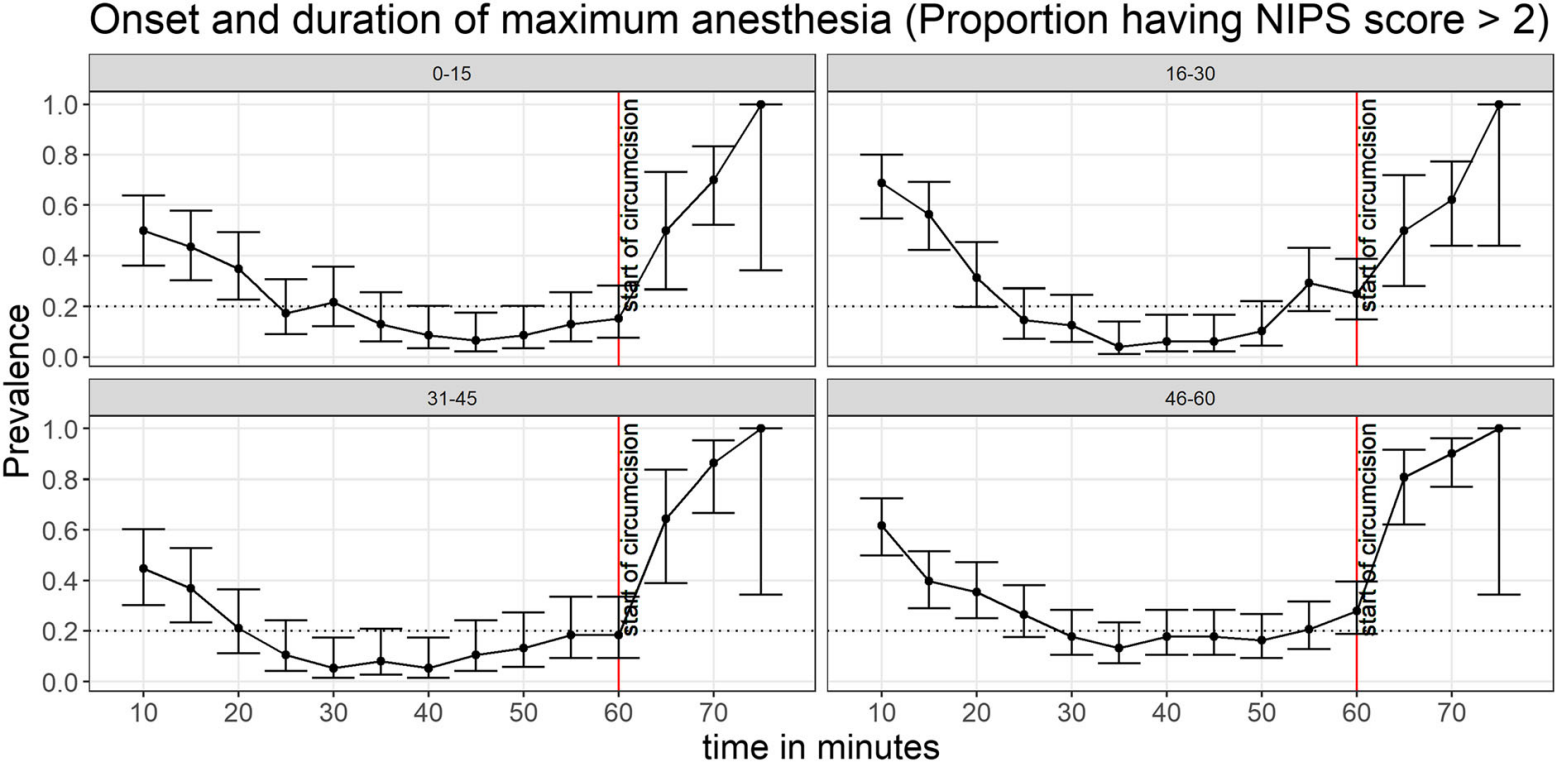
Proportions with NIPS score > 2 following application of topical anaesthetic cream, stratified by age. The error bars represent 95% confidence intervals.

Overall (all ages combined), NIPS scores > 2 were detectable in a few infants (<20%) after at least 30 min following topical anaesthetic application, with maximum anaesthesia lasting <25 min (Figure 2). Stratifying by age, maximum anaesthesia was not achieved among those aged 46–60 days. The duration of maximum anaesthesia also varied with age, lasting <10, 10, 10, and 0 min among those aged 1–15, 16–30, 31–45, and 46–60 days, respectively (Figure 4).

### 3.5 Variation in median NIPS scores (secondary analyses)

Similar patterns to those reported above were observed, with minimum scores recorded before 60 min after topical cream application. Overall, a median score of 0 was achieved between 25 and 55 min (Figure S1). Stratifying by age, the median score of 0 was achieved between 30 and 60, 25–50, 20–55, and 25–40 min in infants aged 0–15, 16–30, 31–45, and 46–60 days, respectively (Figure S2).

## 4 DISCUSSION

While several studies have measured pain control in infants undergoing circumcision,^12^ literature on the optimal timing of when to circumcise is rare—particularly for infant circumcision under topical anaesthesia. Most circumcisions under topical anaesthesia use the 60‐min mark currently recommended in manufacturer instructions and the WHO.^6^ It is possible that inadequate pain control reported in some studies using topical anaesthesia may be due to inappropriate timing of the circumcision. Here, we observed that pain was reasonably controlled after at least 25 min following application of topical anaesthesia, with the effect lasting at least 20 min. The pain was nearly eliminated in all infants under 46 days of age after at least 30 min following application of topical anaesthesia, with the effect lasting up to 10 min. The failure to virtually eliminate pain in older infants may be due to either (i) more sensitivity from increased innervation of the foreskin or (ii) increased behavioural/emotional responses to touch, as well as unfamiliar persons or environments.

Even though some confidence intervals tended toward zero prevalence, none exactly overlapped zero prevalence. This suggests that infant circumcision under topical anaesthesia may not be a completely pain‐free procedure. However, we believe adequate pain control during infant circumcision under topical anaesthesia could be achieved if: (i) infants circumcised are under 46 days of age, (ii) circumcision is started after 25 min following administration of the topical anaesthetic, and (iii) the procedure is completed quickly within about 10 min. Under these conditions, the benefits of infant circumcision outweigh other rare and minor potential harms, as previously suggested by others.^15^ Also, a shorter waiting time may be efficient for mass device‐based circumcision.

The bimodal distribution of baseline responses to pain stimuli (i.e., before anaesthesia takes effect) was an unexpected finding. A transition seems to happen around 40 days of age that results in relatively lower pain scores around that time, which subsequently reverses. We hypothesize this may be due to changes in blood supply to the foreskin and its thickness, resulting in slower wearing off of the anaesthesia, which subsequently reverses.

In the analyses stratified by age, unlike in pooled analyses, the numbers are smaller therefore we are underpowered to conclusively detect pain in at least 20% of infants. However, the patterns observed in these age stratifications could be confirmed in future studies with larger sample sizes.

To our knowledge, this is the first study to report on the variability of pain responses in infants with respect to age and time after applying a topical anaesthetic and to suggest an earlier timing and optimal window of circumcision after application of topical anaesthetics during infant circumcision. The hypotheses generated, including actual mechanisms, may be explored in future studies.

## DISCLOSURE OF INTEREST

All authors have no conflicts of interest to disclose.

## AUTHOR CONTRIBUTIONS

**Stephen Kiboneka:** Conceptualization (Equal); project administration (Lead); writing – original draft (Supporting). **Aggrey Anok:** Data curation (Lead); formal analysis (Supporting). **Regina Nakabuye:** Investigation (Equal). **Silas Odiya:** Investigation (Equal). **Julius Magembe:** Investigation (Equal). **Rose Nazziwa:** Investigation (Equal). **Charles Ddamulira:** Investigation (Equal). **Andrew Mulooki:** Investigation (Equal). **Ronald Moses Galiwango:** Writing – review and editing (Equal). **Stephen Watya:** Supervision (Lead); writing – review and editing (Equal). **Philip S. Li:** Funding acquisition (Equal); writing – review and editing (Equal). **Richard K. Lee:** Funding acquisition (Equal); writing – review and editing (Equal). **Ronald H. Gray:** Funding acquisition (Supporting); writing – review and editing (Equal). **Godfrey Kigozi:** Conceptualization (Supporting); funding acquisition (Supporting); writing – review and editing (Equal). **Edward Nelson Kankaka:** Conceptualization (Equal); formal analysis (Lead); methodology (Lead); visualization (Lead); writing – original draft (Lead).

## Supporting information


**Figure S1:** Median NIPS score after application of topical anaesthetic cream, all ages combined. The error bars represent 95% confidence intervals.

**Figure S2:** Median NIPS score after application of topical anaesthetic cream, stratified by age. The error bars represent 95% confidence intervals.
