# Supplementary figures and images for: Timing for maximum anaesthetic effect of topical cream during early infant circumcision (EIC) in Rakai, Uganda
Source: BJUI Compass. 2023 Jan 16;4(4):423–9. doi: 10.1002/bco2.223 (PMC10268568; doi:10.1002/bco2.223)

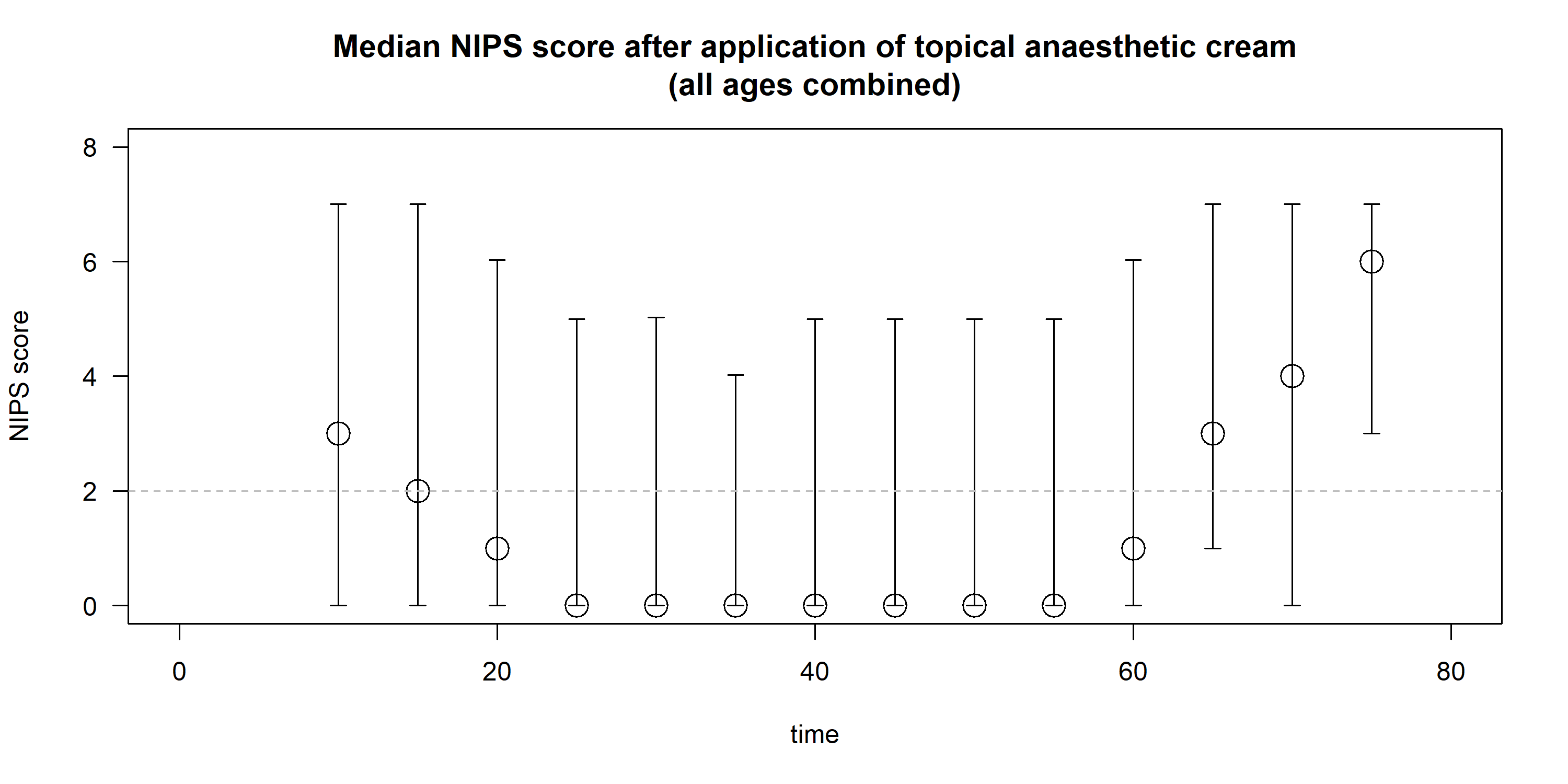

Supplement: Supplementary file 1 — Figure S1: Median NIPS score after application of topical anaesthetic cream, all ages combined. The error bars represent 95% confidence intervals. [file BCO2-4-423-s001.png]

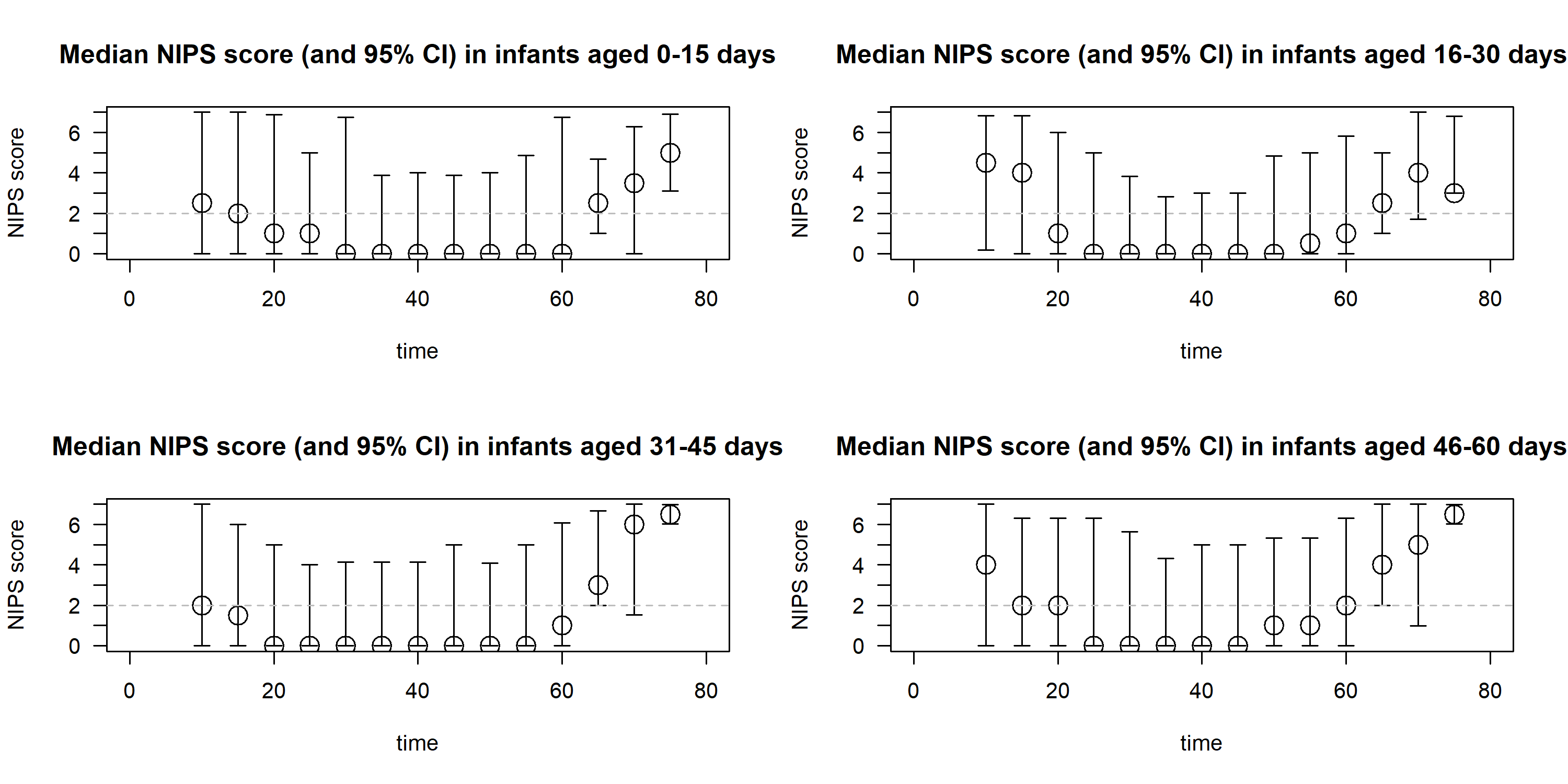

Supplement: Supplementary file 2 — Figure S2: Median NIPS score after application of topical anaesthetic cream, stratified by age. The error bars represent 95% confidence intervals. [file BCO2-4-423-s002.png]
